# VCA supercooling in a swine partial hindlimb model

**DOI:** 10.1038/s41598-024-63041-8

**Published:** 2024-06-01

**Authors:** Yanis Berkane, Irina Filz von Reiterdank, Pierre Tawa, Laura Charlès, Marion Goutard, Antonia T. Dinicu, Mehmet Toner, Nicolas Bertheuil, Aebele B. Mink van der Molen, J. Henk Coert, Alexandre G. Lellouch, Mark A. Randolph, Curtis L. Cetrulo, Korkut Uygun

**Affiliations:** 1 Vascularized Composite Allotransplantation Laboratory, Massachusetts General Hospital, Harvard Medical School, Boston, MA USA; 2 Shriners Children’s Boston, Boston, MA USA; 3 Department of Plastic, Reconstructive and Aesthetic Surgery, Hôpital Sud, CHU Rennes, University of Rennes, Rennes, France; 4 SITI Laboratory, UMR INSERM 1236, Rennes University Hospital, Rennes, France; 5 Department of Plastic, Reconstructive and Hand Surgery, University Medical Center Utrecht, Utrecht, The Netherlands; 6 Center for Engineering for Medicine and Surgery, Department of Surgery, Massachusetts General Hospital, Harvard Medical School, 50 Blossom Street, Boston, MA 02114 USA; 7 Innovative Therapies in Haemostasis, INSERM UMR-S 1140, University of Paris, 75006 Paris, France

**Keywords:** Translational research, Preclinical research

## Abstract

Vascularized composite allotransplantations are complex procedures with substantial functional impact on patients. Extended preservation of VCAs is of major importance in advancing this field. It would result in improved donor-recipient matching as well as the potential for ex vivo manipulation with gene and cell therapies. Moreover, it would make logistically feasible immune tolerance induction protocols through mixed chimerism. Supercooling techniques have shown promising results in multi-day liver preservation. It consists of reaching sub-zero temperatures while preventing ice formation within the graft by using various cryoprotective agents. By drastically decreasing the cell metabolism and need for oxygen and nutrients, supercooling allows extended preservation and recovery with lower ischemia–reperfusion injuries. This study is the first to demonstrate the supercooling of a large animal model of VCA. Porcine hindlimbs underwent 48 h of preservation at − 5 °C followed by recovery and normothermic machine perfusion assessment, with no issues in ice formation and favorable levels of injury markers. Our findings provide valuable preliminary results, suggesting a promising future for extended VCA preservation.

## Introduction

The preservation of transplanted organs has evolved rapidly in the last decade. Static cold storage has given way to the perfusion machine for several solid organs, so much so that it has become the clinical standard in some centers^1–5^. The objective is to improve the quality of transplanted organs by reducing the ischemic damage and ischemia–reperfusion injuries^6–8^ that lead to increased inflammation and a heightened host immune response. An even more ambitious goal is to optimize perfusion extended to several days, with multiple potential clinical applications: Extended preservation would allow for improved cross-matching between donor and recipient by enabling long-distance transport^9–11^, potentially to the other side of the globe. In addition, some procedures require prolonged preparation of the recipient, as is the case with immune tolerance protocols that have shown encouraging results, mainly through bone marrow mixed-chimerism^12–14^. From a vascularized composite allotransplantation (VCA) perspective, these technologies present a tremendous opportunity since the contribution of VCAs to the patient is mainly functional, bringing a debate on the benefit-risk ratio of long-term immunosuppression^15^. Moreover, since VCAs are highly immunogenic due to the presence of a cutaneous component^16^, and particularly sensitive to ischemia due to the muscle component^17,18^, the improvement of VCAs preservation is an essential research topic in the field of reconstructive transplant.

As a potential solution, supercooling techniques have recently demonstrated promising outcomes in solid organ preservation. Supercooling leverages sub-zero storage temperature aimed at minimizing cell metabolism and saving energy and ATP consumption but avoids injuries due to phase change and ice formation^19^. As conventional static cold storage at 4 °C leads to significant injuries after 6 h preservation^20^, these perspectives lead to the potential of extending current storage durations from a few hours to several days. Supercooling prevents ice nucleation using cryoprotection solutions while reaching moderately negative temperatures (− 2 to − 10 °C). Successful preservation of the liver for up to 4 days was demonstrated in a supercooled state^21,22^, and horizontal translation to rodent limbs was shown by our group^23^, but scale-up to large animals remains to be done.

In this work, to evaluate the feasibility and challenges of VCA preservation by supercooling, an initial study with porcine partial hindlimbs was performed. The results were compared with cold static storage, which remains the gold standard in VCA preservation^8^.

## Material and methods

### Animal experiments

Bilateral partial hindlimb harvesting was performed in six 30 kg Female Yorkshire pigs (CBSET Inc, Lexington, MA) for these experiments. All animals were housed in the local Center for Comparative Medicine facility and received humane care in accordance with the National Institutes of Health Guide for the Care and Use of Laboratory Animals^24^. The local Institutional Animal Care and Use Committee (IACUC—protocols 2019N000176 and 2022N000167) and the Animal Care and Use Review Office (ACURO) approved all animal protocols. The authors followed the Animal Research: Reporting of In Vivo Experiments (ARRIVE) guidelines.

### VCA procurement

**Figure 1:**
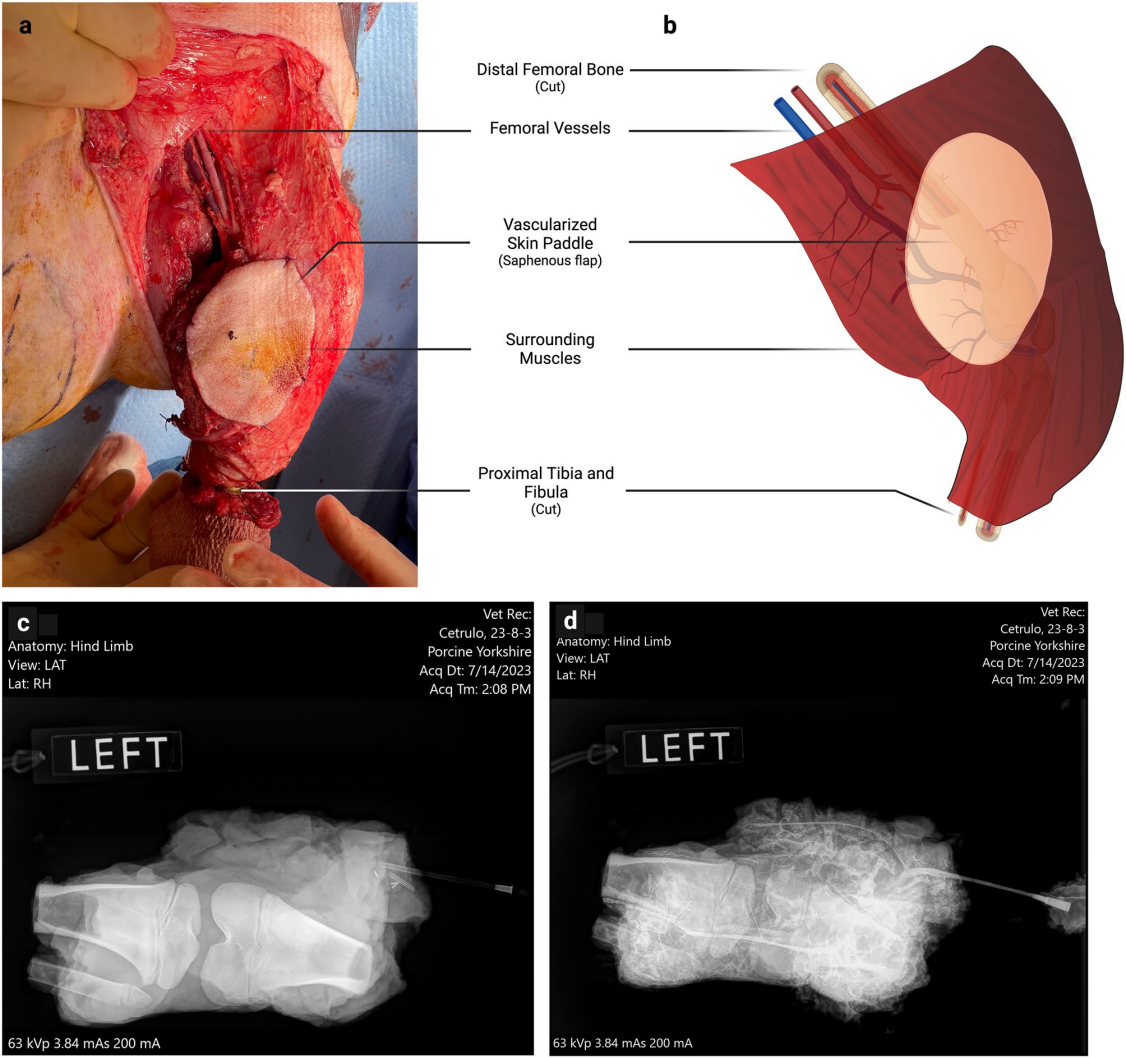
Surgical model of the porcine VCA: partial hindlimb. (**a**,**b**) The partial hindlimb is harvested with the femoral vessels, distal femur, knee joint, and proximal tibia, and fibula bones. The skin paddle consists of an axial saphenous flap, vascularized by the saphenous pedicle, directly derived from the femoral vessels. This allows robust vascularization of the skin paddle. A careful dissection of the hindlimb muscles allows for conserving only deep muscle groups, ensuring complete vascularization of the VCA. Intraoperative bleeding from the bone is confirmed before control using bone wax and division of the vessels. (**c**) Pre- and (**d**) Post-injection iohexol angiography showing adequate vascularization of the harvested muscle groups.

The surgery was performed under general anesthesia (Isoflurane 2–3% inhalation) and orotracheal intubation. All animals were euthanized at the end of the procedure (Pentobarbital/Phenytoin 100 mg/kg IV injection) following the recommendations of the local IACUC and ACURO guidelines. All surgeries were performed by the same surgical team. The VCA model used was a partial hindlimb model previously described^25^. It comprises a composite graft including the distal femoral and proximal tibial and fibular bones, knee joint, surrounding muscles, and a skin paddle corresponding to a saphenous axial fasciocutaneous flap (Fig. 1a,b). This partial hindlimb is vascularized by the femoral vessels, allowing for adequate vascularization of the entirety of the surrounding muscles, as confirmed by angiography (Fig. 1c,d). Before pedicle division, a full dose of 100 UI/Kg of heparin was injected intravenously. After the division of the vessels, the graft was flushed with 50 to 150 ml of cold heparin-saline solution (5%) until clear venous outflow. The VCA then received either static cold storage or supercooling loading.

### Study design

**Figure 2:**
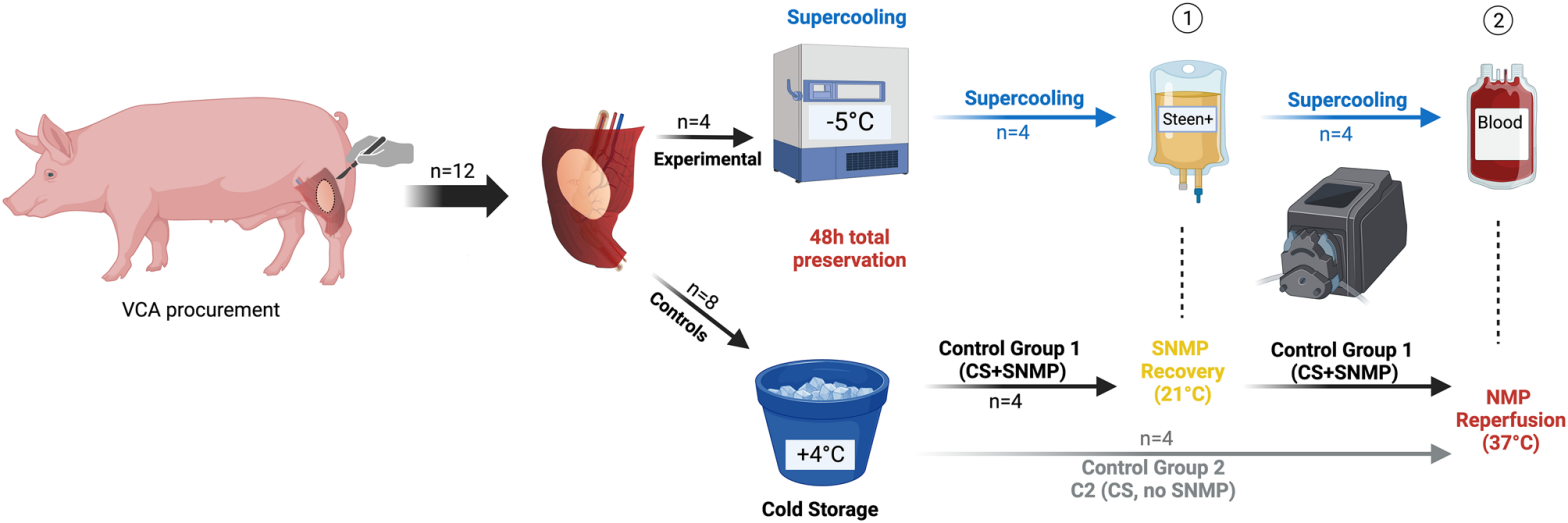
Experimental design. Four hindlimbs were included in each of the three groups undergoing 48 h preservation and recovery. The experimental group underwent supercooling at – 5 °C followed by (1) 2 h SubNormothermic Machine Perfusion (SNMP) recovery and (2) 2 h Normothermic Machine Perfusion (NMP). The control groups 1 and 2 underwent 48 h static cold storage (4 °C, in HTK). Cold Storage + SNMP group received 2 h SNMP before 2 h normothermic reperfusion, while Cold Storage group directly received NMP. All groups received the same monitoring of weight gain, perfusion parameters, biochemical measurements, and histology at each step. HTK: Histidine-Tryptophan-Ketoglutarate solution.

The objective was to assess the supercooling storage of VCA for extended preservation. The total preservation duration was 48 h. One experimental group (Supercooling, “SC group”) and two control groups [(Control group 1: Cold Storage (CS) followed by SubNormothermic Machine Perfusion (SNMP) recovery, or “CS + SNMP group”) and (Control group 2: Cold Storage, or CS group)] were composed. The objective of both control groups was to separately assess the effects of Supercooling and SNMP, which is used to recover Supercooled limbs in this protocol. Each group was comprised of n = 4 replicates (Fig. 2).

### Machine perfusion system

**Figure 3:**
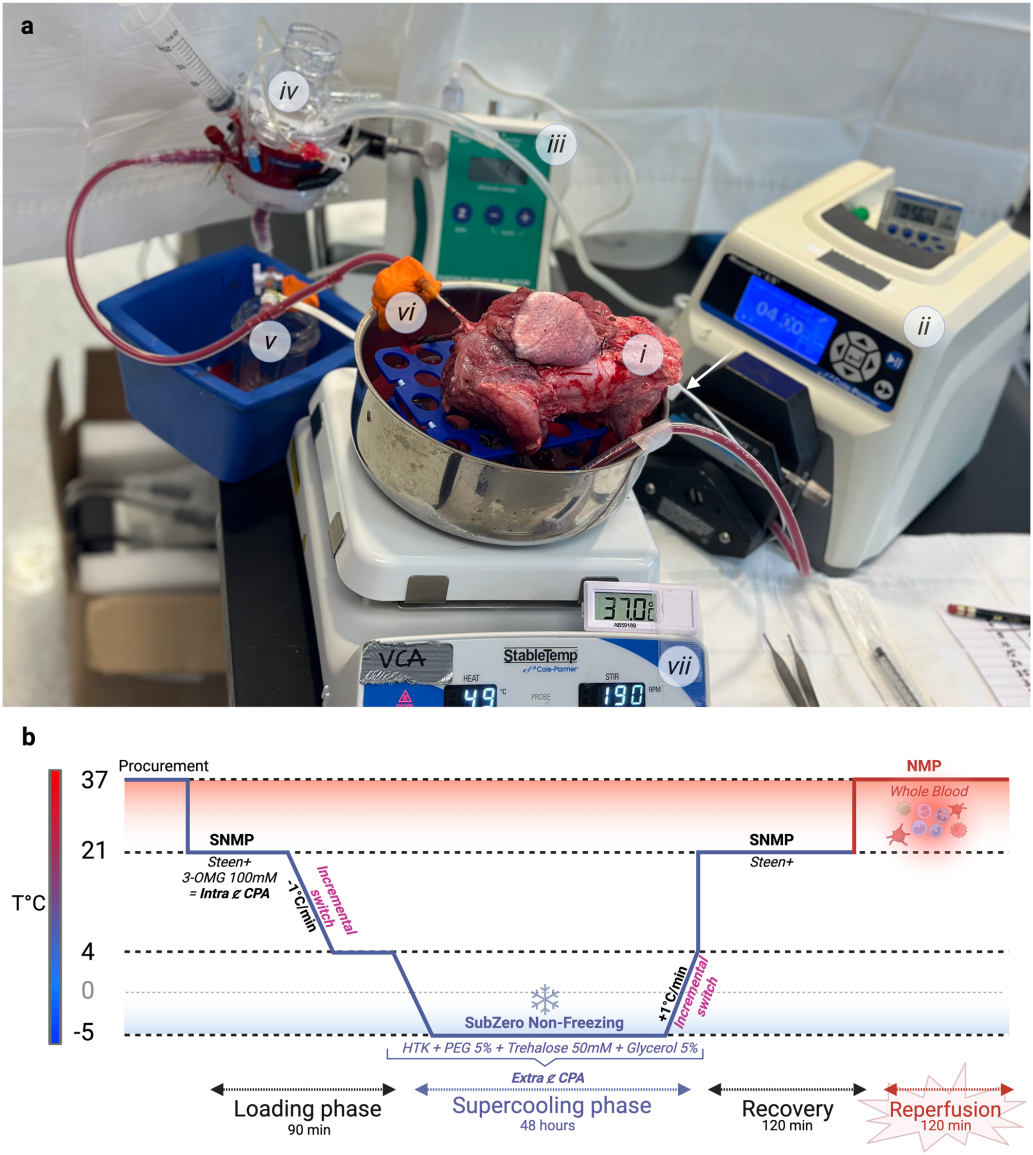
Perfusion system used for loading and recovery, and detailed supercooling protocol. (**a**) Perfusion system. (**i**) The VCA is placed on a rack inside a stainless-steel bowl containing the perfusate solution. (**ii**) A peristaltic pump generates a flow in the silicon tubing to reach (**iv**) the Hollow-fiber membrane oxygenator, which also traps potential air bubbles. (**v**) A pressure sensor is connected to the closed system and to (**iii**) a pressure monitor, allowing for continuous monitoring. (**vi**) The oxygenated perfusate reaches the VCA through the arterial cannula. (**vii**) A hot plate stirrer keeps the perfusate at the desired temperature. White arrow = thermometer. (**b**) Supercooling protocol. The VCA is first loaded with 3-OMG as an intracellular CPA using SNMP (21 °C), before incremental switching to the extracellular CPA cocktail while decreasing the temperature until reaching 4 °C. The limb is then placed for 48 h in a sterile bag full of the extracellular CPA cocktail at − 5 °C in a cooler. The recovery is performed using SNMP with normal Steen + solution with incremental unloading of the extracellular CPA during gradual rewarming. The SNMP is pursued 2 h before switching to whole autologous blood warmed at 37 °C to perform the 2 h NMP phase. *3-OMG* 3-O-methyl-glucose, *CPA* cryoprotective agent, *HTK* histidine-tryptophan-ketoglutarate, *NMP* normothermic machine perfusion, *PEG* polyethylene glycol, *SNMP* subnormothermic machine perfusion.

A custom-made perfusion system was used for subnormothermic machine perfusion for cryoprotective agent loading and recovery and normothermic reperfusion using whole blood. The system was composed of a roller pump (Drive Masterflex L/S, Cole-Parmer, Vernon Hills, IL), a hollow-fiber oxygenator (Affinity Pixie Fusion, Medtronic, Dublin, Ireland), platinum-cured silicon tubings, a pressure sensor, and a pressure monitor (Fig. 3a).

A hot plate stirrer (StableTemp, Cole-Parmer, Vernon Hills, IL) was used for blood warming (37 °C, monitored by a thermometer) and stirring to avoid sedimentation.

### 48-h ex vivo preservation

#### Static cold storage (SCS)

VCAs undergoing SCS were flushed with 50 ml of 4 °C Histidine-tryptophan-ketoglutarate (HTK) solution and placed in a sterile bag with 600 ml of the same solution and transported on ice to a cold room (+ 4 °C), protected from the light. The Cold Storage + SNMP group (CS + SNMP group, SCS with SNMP recovery) was preserved in SCS for 48 h before receiving normothermic machine perfusion with whole blood. The Cold Storage group (CS group, SCS with no SNMP recovery) was preserved for 48 before receiving subnormothermic machine perfusion (SNMP) for 2 h.

#### Supercooling

The supercooling protocol was inspired by our previously detailed work in livers^21^, with the key differences being the cryoprotective agent (CPA) compositions and the perfusate solution used for organ recovery. Partial hindlimbs in the Supercooling Group (SG) were transported on ice to the perfusion system for CPA loading (Fig. 3b). The first phase of the CPA loading consisted of 3-O-methyl-glucose (3-OMG) loading for intracellular cryoprotection. This was performed by 1-h SNMP of 350 ml Steen + solution with 100 mM of 3-OMG. At 60 min, the temperature was lowered to + 4 °C (− 0.5 °C per minute and the loading solution was incrementally switched to a cocktail storage solution optimized in rodent VCA supercooling^23^, based on HTK, Trehalose (50 mM), Polyethylene Glycol (PEG) (5 g) and glycerol (5%). Upon reaching hypothermic temperatures, the flow was lowered to maintain appropriate pressures (30 to 40 mmHg). At 90 min, the VCA was detached from the perfusion system and submerged in 150 mL of storage solution composed of the same Cryoprotective Agents (CPAs). To minimize the risks for ice nucleation, the air–liquid interface was suppressed^26^ by sealing the storage bag before submersion in refrigerant at − 5 °C for 48 h storage.

### SNMP recovery

To avoid ice formation, the VCA was rewarmed to + 4 °C in a water bath at + 37 °C for 13 min based on preliminary studies (Supplementary Fig. 1). The perfusion was initiated at + 4 °C with modified Steen (Steen +)^27^. Upon connection of the organ, the temperature was gradually increased to + 21 °C (mean of + 1.3 °C per minute) and continued for 2 h. In the Supercooling Group, the CPA cocktail solution was flushed out using incremental switch to Steen + , during the gradual rewarming.

### Normothermic reperfusion

To better assess the optimization of the VCA preservation, Normothermic Machine Perfusion (NMP) (37 °C) was performed using autologous whole blood. At the end of the limb harvesting procedure, 500 mL blood was obtained per limb and was stored at + 4 °C in citrate phosphate dextrose adenine (CPDA) bags. After 2-h SNMP (in SC and CS + SNMP GROUP) or directly after rewarming (in CS GROUP), the temperature of the system was increased to 37 °C, and the perfusate was replaced by autologous porcine whole blood which was circulated for 2 h.

### Histology

Biopsies were sampled initially (t = 0) before preservation, at the end of the preservation (t = EOP), of the SNMP recovery (t = EOSR), and of the normothermic blood perfusion (t = EOB). The muscle was biopsied (surgical biopsies) at each time point, including three muscle samples per VCA at the end of the NMP. Skin biopsies were procured initially and at the end of the NMP phase. Samples were fixed in 10% buffered formalin and sent to the pathology department for paraffin inclusion, slicing, and H&E staining. A double-blinded reading was performed, and a muscle injury score was used (Kruit et al.^28^) to compare the groups and the changes over time. For the final muscle samples, a mean score was calculated from the three values.

### Statistical analysis

Quantitative data were entered into Microsoft Excel (Redmond, WA, U.S.A.) for recording, and GraphPad Software's Prism 9 (La Jolla, CA, U.S.A.) was used for all statistical analyses. The significance level, or alpha risk, was set at 5%. A double-sided ANOVA test (if no missing values) or a mixed-effects model (in case of missing values) with the Geisser-Greenhouse correction was performed to compare groups during each recovery phase. Mann–Whitney *U* tests were performed to compare pathology scores at each time point.

All figures are original and were created by the authors, using photographs taken by the authors during the experiments. Diagrams were created using Biorender.com.

## Results

The 12 hindlimbs were equally distributed to the three groups (n = 4 per group): Supercooling (SC group), Cold Storage + SNMP recovery (CS + SNMP group), and Cold Storage without SNMP recovery (CS group). The overall mean initial weight of the VCAs was 475.18 g. The procurement-to-storage time was immediate in the control groups (CS + SNMP and CS groups) and 10.2 + /– 7.8 min in the SC group (time-to-SNMP loading).

### SNMP parameters

Prior to comparing the outcomes for each group, we will briefly summarize the initial step of loading CPAs during machine perfusion protocol, which is performed for only a single group.

#### Loading (supercooling group)

The mean initial loading flow (Supplementary Fig. 2) in this group was 17.5 + /– 2.9 ml/min (at 37 °C) and was increased to reach 31.3 + /– 13.8 ml/min after 30 min. After 60 min (temperature decrease and incremental switch to the extracellular CPA with higher viscosity), the increasing resistances led to a decrease in the flow to 9.2 + /– 7.2 ml/min.

#### Recovery (supercooling and CS + SNMP groups)

**Figure 4:**
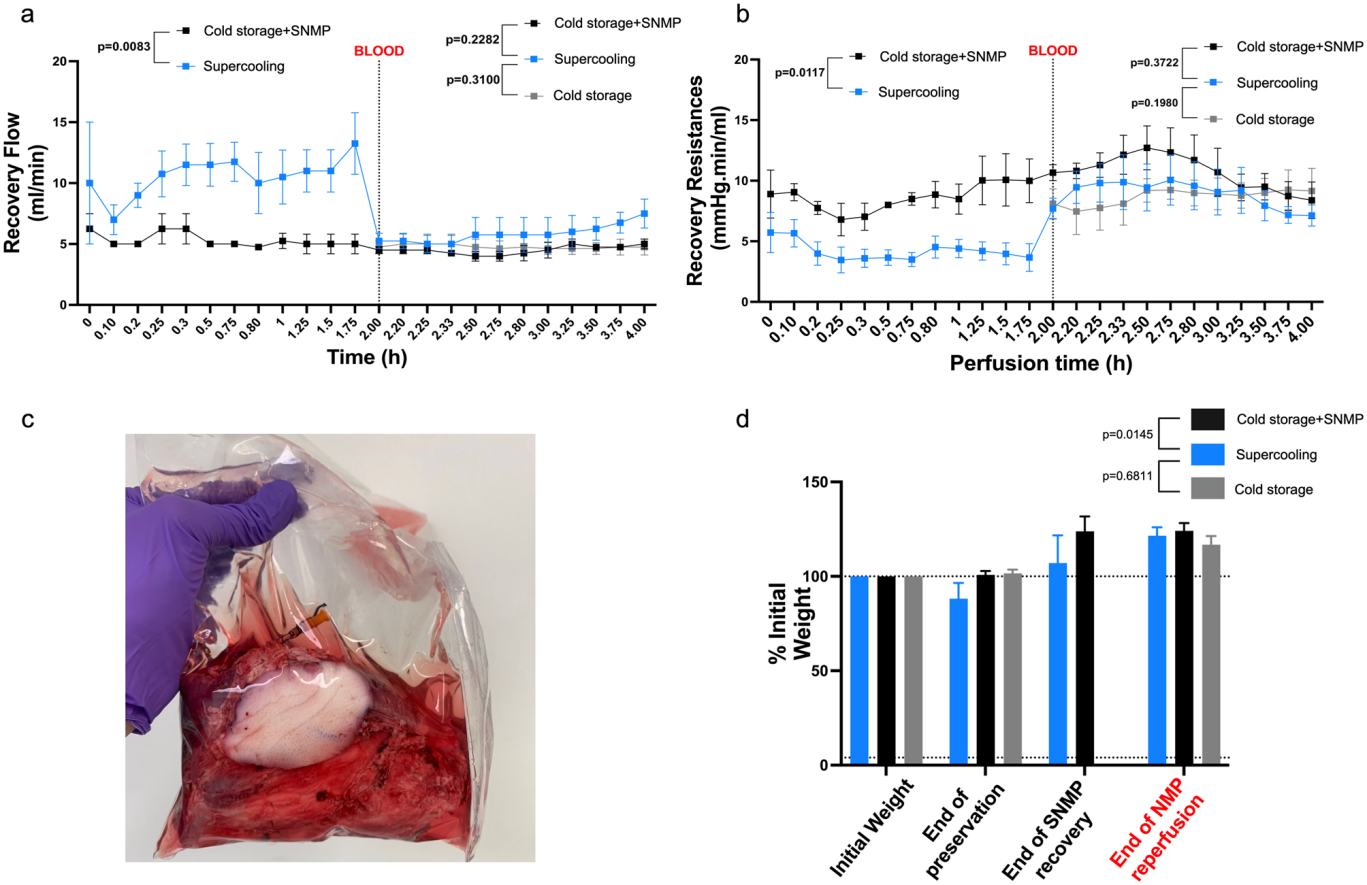
Perfusion parameters of recovered porcine VCAs following 48 h preservation. At t = 2 h, the line marks the perfusate switch to whole blood at 37 °C. (**a**) Flow rate profile during SNMP and NMP recovery showing a significantly higher flow allowed by (**b**) significantly lower vascular resistance in the SC group during the SNMP phase. No significant difference was found between the three groups in the NMP phase. Note: A mixed-effects model with the Geisser-Greenhouse correction was performed to compare groups during each phase. (**c**) Weight gain following SNMP and NMP was significantly lower in the SC group compared to CS + SNMP GROUP but not with CS GROUP. (**d**) Aspect of the stored VCA following 48 h supercooling, showing the absence of ice nucleation.

Following 48 h of storage and after 5 min of adaptation to the pressure, the mean initial flow during (Steen +)-based SNMP was 7.0 + /– 2.45 and 5.0 + /– 0.0 ml/min in the Supercooling group and the CS + SNMP group, respectively (p = 0.008, Fig. 4a). The corresponding vascular resistance (Fig. 4b) was calculated to be 5.66 + /– 2.27 and 9.05 + /– 1.41 mmHg.min/ml, respectively (p = 0.011). The supercooled limbs (Fig. 4c) tended to lose weight following loading and storage with a mean weight at 48 h of 88 + /– 8.26% of the initial weight *versus* 100.79 + /– 2.05% in CS + SNMP group (p = 0.11) and 101.57 + /– 1.99 in Cold storage group (p = 0.11). At the end of the SNMP recovery, weight gain was observed in Supercooled limbs (107.03 + /– 8.26%) and CS + SNMP group (123.87 + /– 7.81%) with no statistically significant difference (p = 0.11, Fig. 4d).

**Figure 5:**
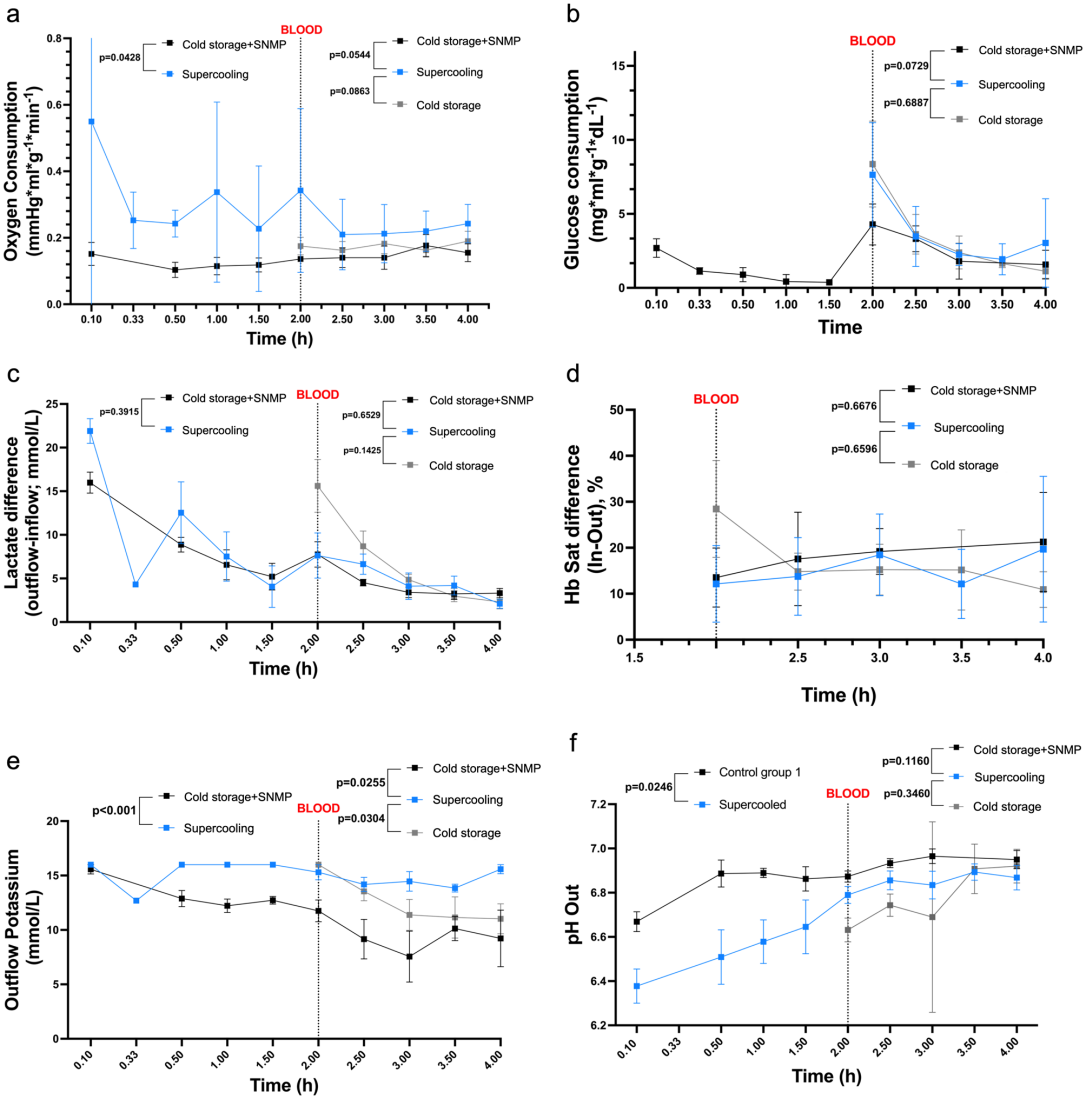
Perfusate analysis during recovery. (**a**) Oxygen consumption was higher in the Supercooled group during recovery versus control, but the difference was only significant in the SNMP phase. (**b**) The glucose consumption was not measurable during the SNMP recovery due to interference with the 3OMG. The supercooled limbs seemed to consume more glucose in the NMP phase versus Cold Storage + SNMP group, but the difference was not significant. (**c**) Lactate release levels were comparable between groups during both phases. (**d**) Hemoglobin arteriovenous difference was comparable between groups during the NMP phase. (**e**) Potassium release was significantly higher in the Supercooled group during the SNMP phase. (**f**) The pH was lower in the Supercooled group during the NMP phase and tended to reach similar values as control groups during the NMP phase.

During the 2 h SNMP period, the oxygen consumption was higher in the Supercooled group with 0.34 + /– 0.24 *versus* 0.136 + /– 0.01 mmHg*ml*g^–1^*min^–1^ in the CS + SNMP group (p = 0.02) at t = 2 h (p = 0.043 for the SNMP phase, Fig. 5a). Mean glucose consumption in the CS + SNMP group was 2.69 + /– 0.63 and 0.38 + /– 0.19 mg*ml*g^–1^*dl^–1^ at t = 0 and t = 2 h SNMP, respectively (Fig. 5b). Glucose consumption in the Supercooled group could not be interpreted because of interference between Glucose and 3-OMG in the biochemical analyzer during this phase.

The pH was lower in the Supercooling group with a mean of 6.38 + /– 0.16 and 6.79 + /– 0.08 initially and at the end of SNMP, versus 6.67 + /– 0.09 and 6.87 + /– 0.11 in CS + SNMP group (p = 0.025, Fig. 5f). Similarly, the lactate release (Fig. 5c) was initially higher in the Supercooling group (21.9 + /– 2.83 mmol/L) when compared with Cold Storage + SNMP group (15.99 + /– 2.39 mmol/L, p = 0.06) but reached similar values at the end of the SNMP recovery (7.63 + /– 5.16 and 7.76 + /– 2.93 mmol/L, respectively). The potassium concentration (Fig. 5e) in the outflow was higher in the Supercooled group during the whole SNMP phase (p < 0.001).

### NMP parameters (whole blood, all groups)

Blood viscosity imposed higher vascular resistance during NMP than SNMP. Mean initial vascular resistance was 7.71 + /– 1.74, 10.66 + /– 1.34, and 8.10 + /– 1.23 mmHg.min/ml in the Supercooled, CS + SNMP and CS groups, respectively (p = 0.03 and 0.54). At the end of the NMP phase, the resistance in the experimental group was still lower without reaching statistical significance (p = 0.372 and 0.198) with a higher flow (7.5 + /– 2.3 versus 5.0 + /– 0.81 and 4.75 + /– 0.65 ml/min). Weight gain following 2 h-NMP was comparable in all 3 groups (121.52 + /– 4.45, 124.11 + /– 4.12 and 116.79 + /– 4.57%, respectively).

Blood perfusion allowed for better correction of the pH with values of 6.87 + /– 0.11, 6.95 + /– 0.08, and 6.92 + /– 0.08 at the end of the perfusion. Similarly, the lactate release decreased progressively in all groups to reach its lowest at the end of the NMP phase, with insignificantly lower values for the Supercooled group. The outflow potassium concentration was higher in the Supercooled group than in both controls with statistical significance. The oxygen consumption was significantly higher in the Supercooling group (p < 0.001) compared to both the control groups. The glucose consumption was also higher in the experimental limbs without reaching statistical significance (p = 0.073 and 0.689).

The hemoglobin arteriovenous difference at the end of the 2 h-NMP was 19.68 + /– 15.9, 21.27 + /– 10.77, and 10.93 + /– 3.90% in the Supercooling, CS + SNMP and CS groups, respectively, with no statistical significance (Fig. 5d). 

### Histology

**Figure 6:**
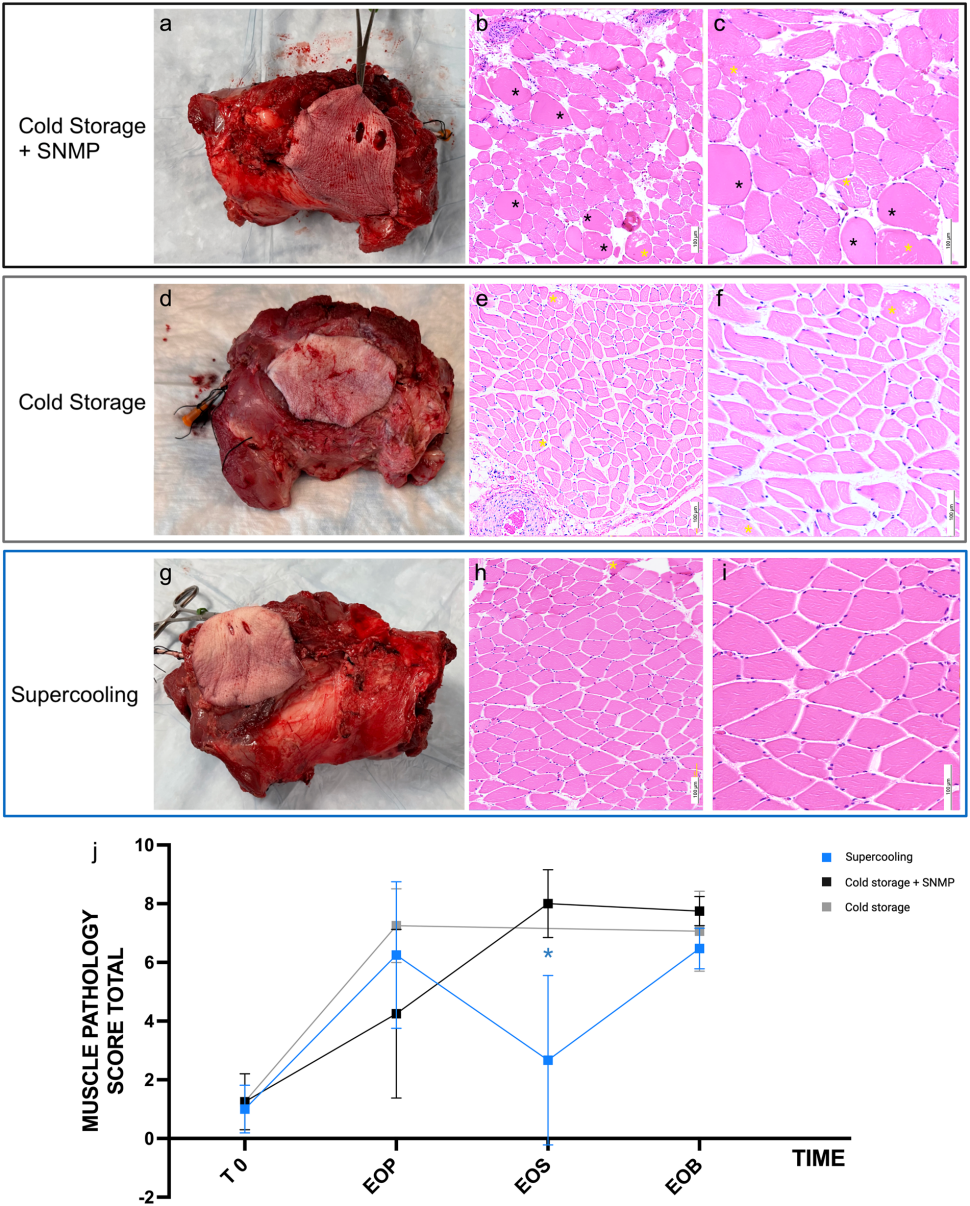
Macroscopic and Microscopic results on the porcine VCAs following 48 h preservation and recovery. (**a**,**d**,**g**) The macroscopic aspect of the grafts following 2 h of 37 °C NMP with whole blood was similar in the Cold Storage + SNMP, Cold Storage and Supercooling groups, respectively. The skin paddle presented an ecchymotic aspect, which seemed milder in the Supercooling group, but no significant differences were found histologically. (**b**,**c**) At the microscopic level, the Cold storage + SNMP group showed significant interstitial edema, ischemic myocytes (black asterisks) and myocyte fiber injuries (yellow asterisks). (**e**,**f**) The Cold storage group showed major edema and mild fiber architecture disruption. (**h**,**i**) The Supercooling group showed mild interstitial edema and minor muscle fiber injuries when compared with both control groups. (**j**) A blinded microscopic score (Kruit et al. 2021) was performed. The overall scores were compared at several time points for each group: T0 (initial), EOP (end of preservation), EOS (end of Steen + SNMP recovery) and EOB (end of NMP blood recovery). A significantly lower muscle injury score was found between the Supercooling group and the Cold storage + SNMP group at the end of the Steen + recovery. The difference between experimental and control groups was not statistically significant at the other time points. (**b**,**e**,**h**) light microscopy (LM), X100, Hematoxylin and Eosin (H&E) staining; (**c**,**f**,**i**) LM X200, H&E.

Figure 6 presents the macroscopic and microscopic aspects of the VCAs following 48 h of preservation, SNMP recovery (for Supercooling and CS + SNMP groups), and whole blood normothermic machine perfusion. Overall, no macroscopic difference was found between groups, despite milder ecchymoses in the supercooled grafts. When focusing on the myocyte preservation, blinded microscopic assessment of the muscle only showed statistically significant differences with lower injuries at the end of the SNMP phase (Kruit et al. VCA score)^28^. Skin and nerve microscopic analyses showed no significant differences in all three groups following normothermic blood perfusion (Supplementary Fig. 3 and Table 1).

## Discussion

Despite the high immunogenicity of vascularized composite allografts- primarily attributed to the skin component^15,29^- over 140 limb transplants and nearly 50 partial or full-face transplants and successful penile and uterine transplantations^30,31^ have been performed globally^15,32^. In order to combat the high immunogenicity of VCAs, the development of protocols for extended VCA preservation and ex vivo manipulation of the VCA represent critical goals for this burgeoning field. Superior preservation mitigates ischemia–reperfusion injury, which reduces inflammation and the subsequent host immune response and may buy time for better matching of national (and perhaps even international) donor-recipient pairs. Extended preservation also holds the potential for increasing the total number of available organs, by allowing national, transcontinental and international graft transportation as recently demonstrated in solid organs^33^. These perspectives would greatly reduce waiting list delays, improve donor-recipient immune and phototype matching, and avoid complex logistic arrangements such as patient transfers. Implementing immune tolerance through mixed chimerism procedures would necessitate a preparation time frame of 2 to 6 days^12–14^, a requirement that is not possible in brain dead donors (but only living donors, irrelevant for VCA) with current preservation protocols. Additionally, ex vivo manipulation with gene and cell therapies in the VCA during loading and/or recovery has the potential to abrogate rejection mechanisms as well, providing a synergic approach. Consequently, it is imperative to develop novel protocols for extended VCA preservation.

This study demonstrated the first successful supercooling of porcine VCAs for 48 h. Despite the complexity of this model, which is composed of multiple tissue types, no uncontrolled spontaneous ice nucleation was observed during 2 days of subzero preservation. This can be challenging due to the significant volume of the model. Indeed, ice nucleation is a stochastic process and, therefore, increases with preserved tissue volume^26^. Moreover, the composite aspect of the porcine partial hindlimb makes it difficult to suppress the air/liquid interface, which is a critical step to further avoid ice formation^22,26^. The combination of CPAs and the protocol used in our study made it possible to avoid this phenomenon at − 5 °C. Pushing the limits further by reaching lower temperatures could lead to a more significant reduction in cell metabolism, potentially resulting in improved organ recovery and decreased ischemia–reperfusion injuries. Further work should focus on optimizing the balance between deeper negative temperatures and a stable supercooling state.

In the present results, recovery of supercooled VCAs revealed successful restoration of post-preservation vascular flow. Supercooled limbs showed significantly lower vascular resistance during SNMP recovery following 48 h preservation when compared with static cold stored controls. Interestingly, this difference was not significant during the NMP phase. Moreover, the vascular resistance in the CS group was lower than in the CS + SNMP group. This highlights the ambivalence of SNMP, which, on one hand, allows for recovering the limb to a sub-physiological state while clearing toxic metabolites^22^ and, on the other hand, exposes the graft to potential endothelial injuries^34^. More work is needed to balance the role of SNMP in supercooling techniques to reach optimized protocols, ensuring adequate recovery following extended preservation. Weight gain was minor and stayed below 20% at the end of the NMP phase, with no significant difference between groups. However, a significant difference was found between the supercooled and CS limbs at the end of the SNMP, which is consistent with the vascular resistance outcomes. Among the outcome metrics, weight gain and vascular resistance are the earliest and most critical parameters in organ machine perfusion. Meyers et al. demonstrated that weight gain was correlated with microscopic muscle injury and was the earliest evidence of VCA dysfunction during ex*-*vivo normothermic limb perfusion in swine^34^. In addition, vascular resistance is closely linked to endothelial cell injury: shear-stress-induced injuries cause cellular edema, which eventually increases perfusion resistance by external compression of the microvessels^35^. Moreover, no autonomic nerve control is possible during *ex-vivo* perfusion, and circulating vasoactive substances show poor action, leaving the paracrine relaxing factors as the main effector for vasodilatation. These endothelial injuries should, therefore, be estimated using flow parameters such as vascular resistance and weight gain^34–36^. Specifically, assessing endothelial injuries following extended preservation and ex vivo recovery using electron microscopy techniques would be of great interest and should be included in subsequent studies.

Another major finding was the higher oxygen consumption of the supercooled limbs during recovery. This parameter directly indicates higher metabolism, potentially linked to better cell viability permitted by supercooling. This was also suggested by a higher glucose consumption during the NMP phase without reaching statistical significance. The evaluation was done under physiological conditions through whole-blood NMP, which significantly improved the relevance of those metrics. However, if the microscopic assessment of the muscle following NMP suggested better preservation of the myocyte structure and lower edema in the Supercooling group, no significant differences with CS controls were found after this physiological recovery. More replicates in each group could help increase the statistical significance. Alternatively, a longer normothermic phase with fresh blood could have unveiled more IRI and potentially more differences in pathology scoring between groups. NMP as a simulation for physiological replantation has been used by other authors, but to date, there is no consensual protocol^22,37,38^.

However, this last point highlights the first limit of these results: the absence of transplantation with in vivo assessment of the VCA. The 2 h blood NMP phase limits the extrapolation to the in vivo behavior of the graft. However, the relevance of NMP has been demonstrated. It appears to be more ethical regarding animal welfare since it allows for an ex-vivo assessment of graft quality following each preservation technique. Our experience with other preservation protocols showed that transplanting VCAs in a poor metabolic condition can lead to a critical decrease in the recipient animal’s general condition, which needs to be avoided^36^. The purpose of this preliminary study was, therefore, to act as a first phase of *ex-vivo* optimization. The most critical challenge remains upscaling preclinical results to the clinical reality. The model used in our study is composed of a substantial volume of muscle, which is the limiting tissue when focusing on ischemia–reperfusion injuries. In contrast, human upper limb VCA often mainly comprises the distal tendons of the forearm muscle groups, with a limited amount of muscle belly. Similarly, face transplantation involves thin but important muscles. Being able to preserve multiple bulky muscle groups for several days with supercooling, therefore, holds great promise for clinical applications. Nerve function following recovery becomes of crucial interest, and implementing nerve conduction and muscle contraction assessments seems to be one of the most critical next steps.

Moreover, the Supercooling protocol inspired by previous work in the liver showed limitations in this VCA study: the potassium release was significantly higher in the Supercooled limbs for both recovery phases. This could be due to either rhabdomyolysis due to ischemia–reperfusion injuries or due to potassium storage and release from the CPA cocktail. This second hypothesis is more likely: the lactate release was similar in all groups, the histology showed better preservation of the muscle fiber architecture, and the CPA cocktail solution based on HTK (potassium concentration 8–9 mmol/L) was loaded for 30 min using machine perfusion *versus* a hand flush in the control groups. To elucidate on this, it could be of interest to compare the potassium release levels of CPA-stored limbs with VCAs stored in a perfusate solution based on physiologic potassium concentrations, such as Steen +. Media composition has been shown to be critical in VCA machine-perfusion protocols^39^; therefore, current work focuses on optimizing the CPA cocktail and recovery solution compositions.

This study is part of a broader research trend. The recent exponential advancement of perfusion machines in kidney^2,40^, liver^1,4^, heart^3,41^, and lung^42,43^ transplantation hints at potential applications in VCAs. The variety of tissues composing VCAs brings a substantial challenge when translating solid organ preservation protocols. Kruit et al. and later our team described the first machine perfusion protocols applied to large animal VCA^27,28,36,39,44–46^. Nonetheless, current dynamic hypothermic and SNMP techniques are limited to preservation periods of 24 to 36 h due to factors such as edema and vascular resistance^34,47–49^. Supercooling has proven itself a promising technique to overcome these limits and go beyond several days in liver^22,50^. The sub-zero temperature reduction, combined with the absence of ice nucleation, has been shown to extend liver preservation to 96 hours^50^. Hence, developing a relevant VCA supercooling protocol appears worthwhile^51^. We aimed for 48 h of preservation since this duration would make it possible to combine with immune tolerance protocols based on mixed chimerism. Being able to preserve a VCA procured on a brain-dead donor for 2 days would allow preparing the recipient (body irradiation, immunosuppressive regimen) for combined VCA and bone marrow transplantation, as previously described^14,52^. However, while we focused on supercooling cryopreservation, other techniques are of great interest for organ and VCA preservation. Vitrification aims to drastically and quickly decrease the organ temperature to the extreme, reaching a glass-like state^53,54^, while partial freezing seeks to control the ice nucleation and its potentially harmful effects on the tissues^55,56^. These alternative techniques showed promising results in solid organs, and further studies should explore them in VCAs.

This preliminary work showed promising outcomes regarding microvascular preservation estimated by the perfusion parameters and histologic preservation of the myocytes and muscle fibers. However, this protocol, highly inspired by liver supercooling, still needs substantial optimization. In order to address the limits shown in metabolic parameters, adjustment in CPA and media seems necessary before in vivo experimentation of VCA transplantation following prolonged storage, eventually opening the door to a wide range of new applications.

### Supplementary Information

Supplementary Information.

## Data Availability

All data generated and analyzed during this study have been included in this manuscript and its Supplementary Information file. All raw data can be provided on demand by the corresponding author.
