# Supplementary material for: VCA supercooling in a swine partial hindlimb model
Source: Sci Rep. 2024 Jun 1;14:12618. doi: 10.1038/s41598-024-63041-8 (PMC11144209; doi:10.1038/s41598-024-63041-8)
Supplement: Supplementary file 1 — Supplementary Information. [file 41598_2024_63041_MOESM1_ESM.docx]

**Scaling up VCA Supercooling in a Swine Partial Hindlimb Model**

**Supplementary Information**

**Supplementary Figure 1**.

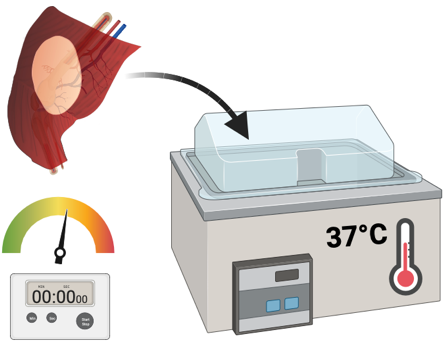


**Caption:**

***Supplementary Figure 1****.* ***Determination of reheating time for porcine partial hindlimbs.*** *The* *partial hindlimb was procured and supercooled at -5°C for at least 48 hours. Upon removal from the chiller, temperature probes were placed in the center of the graft, and the graft was placed in a water bath at 37°C. Temperature recording showed that the graft core reached 0°C at 9m30s, +4°C at 13m and +21°C at 33m30s. Time was rounded up or down to the closest half-minute.*


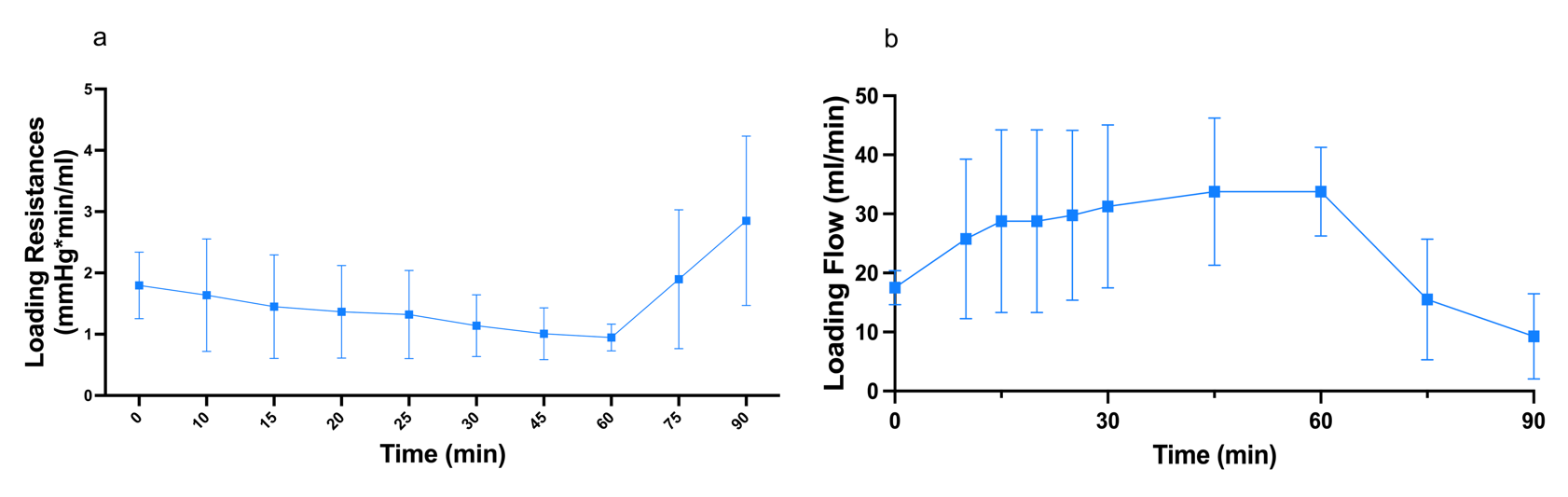
**Supplementary Figure 2***.*

**Caption:**

***Supplementary Figure 2****.* ***Supercooled limbs loading profile****. The vascular resistance was calculated using the formula R=P/Q (R=Vascular Resistance, mmHg.min/ml; P=Measured Pressure, mmHg; Q=Perfusion Flow Rate, ml/min). The decreasing resistance during the loading phase allowed for increasing the flow until t=60 min, where was started the incremental switch from Steen+ to the CPA cocktail solution while decreasing the temperature. The viscosity of the circulating perfusate and the lower temperature led to increasing resistance and, therefore, decreasing the flow rate.*

**Supplementary Figure 3**

**
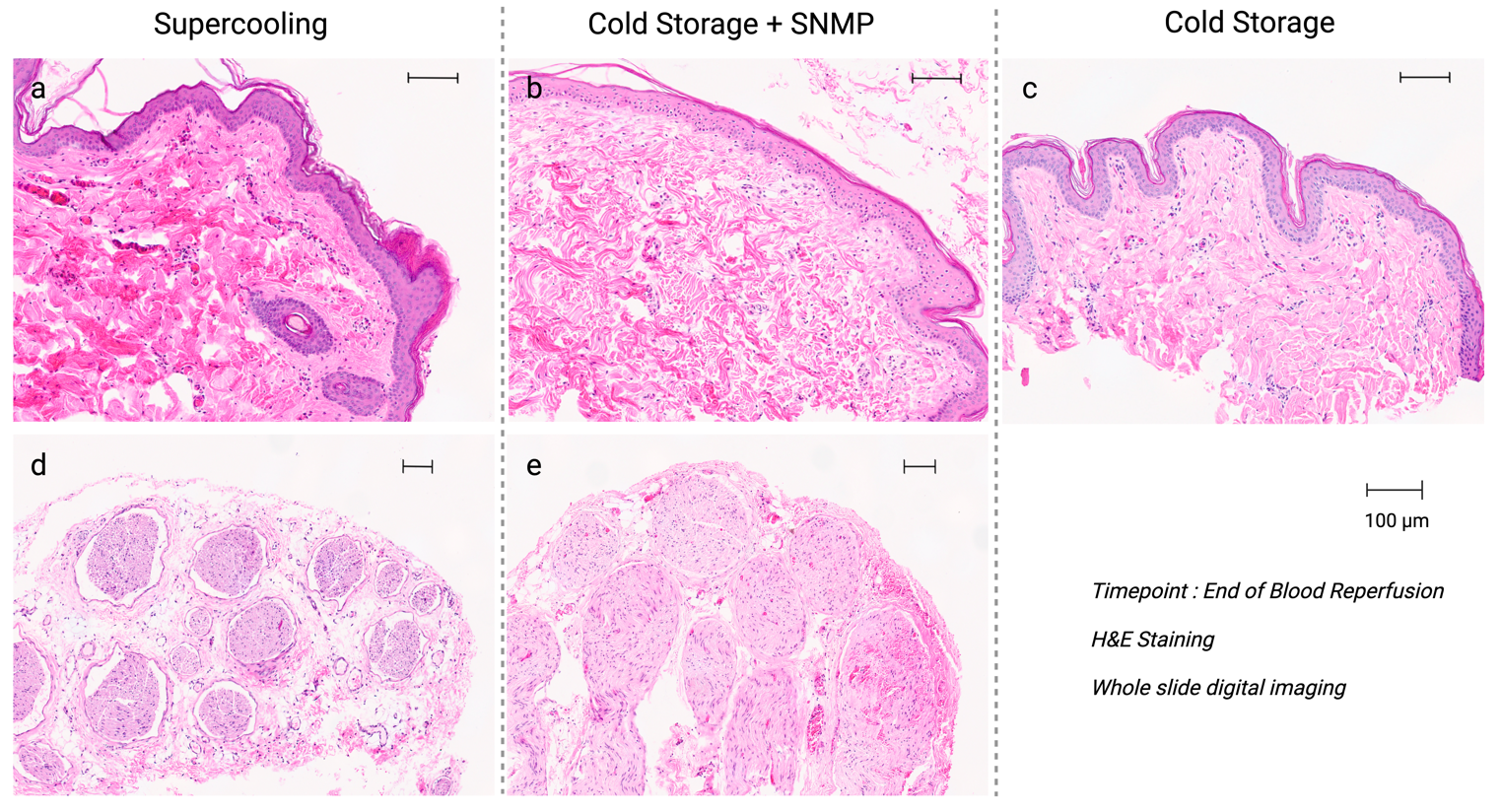
**

**Caption:**

***Supplementary Figure 3. Representative results of histological aspects of skin and nerve tissues following whole blood normothermic perfusion****.* Hematoxylin and Eosin staining, whole slide digital imaging. **a.** Skin biopsy in a supercooled limb following 2h SNMP and 2h NMP. **b**. Skin biopsy in a limb undergoing static cold storage followed by 2h SNMP and 2h NMP. **c.** Skin biopsy in a limb undergoing static cold storage followed by 2h NMP. **d.** Nerve biopsy in a supercooled limb following 2h SNMP and 2h NMP. **e.** Nerve biopsy in a limb undergoing static cold storage followed by 2h SNMP and 2h NMP. No major differences were found between groups in these two tissue types. *Detailed description of the histological analysis can be found in Supplementary Table 1.*

**Supplementary Table 1**

|  |  |  | **SKIN** | | | **MUSCLE/OTHER** |
| --- | --- | --- | --- | --- | --- | --- |
| **Group** | **Timepoint** | **Tissue** | **Banff Grade** | **Description** | **Pathology component score** | **Pathology scoring : Comments** |
| CS | C=EO blood | Skin | 0 | Unremarkable | pc0, pa0, e0, ei0, c0, v0, cv0 |  |
| CS | C=EO blood | Skin | 0 | NDAR | pc0, pa0, e0, ei0, c0, vx, cvx |  |
| CS | C=EO blood | Skin | 0 | Unremarkable | pc0, pa0, e0, ei0, c0, vx, cvx |  |
| CS | C=EO blood | Skin | 0 | Not fixed |  |  |
| CS + SNMP | T=0 | Skin | 0 | NDAR | pc0, pa0, e0, ei0, c0, v0, cv0 |  |
| CS + SNMP | A=EOP | Skin | 0 | NDAR | pc0, pa0, e0, ei0, c0, v0, cv0 |  |
| CS + SNMP | B=EO Steen | Skin | 0 | NDAR | pc0, pa0, e0, ei0, c0, v0, cv0 |  |
| CS + SNMP | C= EO blood | Skin | 0 | NDAR | pc0, pa0, e0, ei0, c0, v0, cv0 |  |
| CS + SNMP | T=0 | Skin | 0 | NDAR | pc1, pa0, e0, ei0, c1, v0, cv0 |  |
| CS + SNMP | A=EOP | Skin | 0 | NDAR | pc0, pa0, e0, ei0, c0, vx, cv0 |  |
| CS + SNMP | B=EO Steen | Skin | 0 | NDAR | pc0, pa0, e0, ei0, c0, v0, cv0 |  |
| CS + SNMP | C= EO blood | Skin | 0 | NDAR | pc0, pa0, e0, ei0, c0, v0, cv0 |  |
| CS + SNMP | A=EOP | Skin | 0 | NDAR | pc0, pa0, e0, ei0, c0, v0, cv0 |  |
| CS + SNMP | B=EO Steen | Skin | 0 | NDAR | pc0, pa0, e0, ei0, c0, v0, cv0 |  |
| CS + SNMP | C= EO blood | Skin | 0 | NDAR; congestion | pc0, pa0, e0, ei0, c0, v0, cv0 |  |
| CS + SNMP | A=EOP | Skin | 0 | NDAR | pc0, pa0, e0, ei0, c0, vx, cv0 |  |
| CS + SNMP | B=EO Steen | Skin | 0 | NDAR | pc0, pa0, e0, ei0, c0, vx, cv0 |  |
| CS + SNMP | C= EO blood | Skin | 0 | NDAR | pc0, pa0, e0, ei0, c0, v0, cv0 |  |
| CS + SNMP | C= EO blood | Nerve |  |  |  | Generally unremarkable |
| CS + SNMP | C= EO blood | Nerve |  |  |  | NDAR |
| Supercooling | A=EOP | skin | 0 | NDAR; congestion | pc0, pa0, e0, ei0, c0, v0, cv0 |  |
| Supercooling | B=EO Steen | skin | 0 | NDAR | pc0, pa0, e0, ei0, c0, v0, cv0 |  |
| Supercooling | C= EO blood | skin | 0 | NDAR | pc0, pa0, e0, ei0, c0, v0, cv0 |  |
| Supercooling | T=0 | Skin | 0 | NDAR | pc0, pa0, e0, ei0, c0, v0, cv0 |  |
| Supercooling | A=EOP | Skin | 0 | NDAR | pc0, pa0, e0, ei0, c0, v0, cv0 |  |
| Supercooling | B=EO Steen | Skin | 0 | NDAR | pc0, pa0, e0, ei0, c0, v0, cv0 |  |
| Supercooling | C= EO blood | Skin | 0 | NDAR | pc0, pa0, e0, ei0, c0, v0, cv0 |  |
| Supercooling | A=EOP | Skin | 0 | NDAR | pc0, pa0, e0, ei0, c0, vx, cv0 |  |
| Supercooling | B=EO Steen | Skin | 0 | NDAR | pc0, pa0, e0, ei0, c0, v0, cv0 |  |
| Supercooling | C= EO blood | Skin | 0 | NDAR | pc0, pa0, e0, ei0, c0, v0, cv0 |  |
| Supercooling | A=EOP | Skin | 0 | NDAR | pc0, pa0, e0, ei0, c0, v0, cv0 |  |
| Supercooling | B=EO Steen | Skin | 0 | NDAR | pc0, pa0, e0, ei0, c0, v0, cv0 |  |
| Supercooling | C= EO blood | Skin | 0 | NDAR | pc0, pa0, e0, ei0, c0, vx, cv0 |  |
| Supercooling | C= EO blood | Skin | 0 | NDAR | pc0, pa0, e0, ei0, c0, v0, cv0 |  |
| Supercooling | C= EO blood | Nerve |  |  |  | Mild edema in nerve bundles |
| Supercooling | C= EO blood | Nerve |  |  |  | Generally unremarkable |

**Caption:**

***Histology analysis by a blinded, experienced pathologist****. EO Blood: End of Whole Blood Normothermic Perfusion; EOP: End of Preservation Period; EO Steen: End of Steen SubNormothermic Machine Perfusion. T=0: Initial (time of the procurement). NDAR: No Diagnostic Abnormality.
NB1: Banff VCA classification (2007) is systematically performed in VCA samples even with no allotransplantation. NB2: Skin pathology component score is derived from Banff 2007 VCA classification.*
